# Family caregiver experiences and needs across health conditions, relationships, and the lifespan: a Qualitative analysis

**DOI:** 10.1080/17482631.2023.2296694

**Published:** 2024-01-11

**Authors:** Sarah A. Neller, Megan Thomas Hebdon, Emily Wickens, Debra L. Scammon, Rebecca L. Utz, Kara B. Dassel, Alexandra L. Terrill, Lee Ellington, Anne V. Kirby

**Affiliations:** a College of Nursing, University of Tennessee, Knoxville, TN, USA; b College of Nursing, University of Utah, Salt Lake City, UT, USA; c College of Nursing, University of Texas, Austin, TX, USA; d College of Social and Behavioral Sciences, University of Utah, Salt Lake City, UT, USA; e David Eccles School of Business, University of Utah, Salt Lake City, UT, USA; f College of Health, University of Utah, Salt Lake City, UT, USA

**Keywords:** Informal caregiving, Qualitative, focus group, family, disability

## Abstract

**Purpose:**

The purpose of this study was to understand the lived experiences of family caregivers who provide care to individuals across a broad range of ages, caregiving relationships, and health conditions and/or disabilities. Family caregiver research is typically siloed by health condition or by caregiving relationship, leaving gaps in understanding similarities and differences among caregivers.

**Methods:**

We hosted three virtual focus groups with diverse family caregivers (*n* = 26) caring for an individual with a long-term disability and/or health condition(s). We conducted a qualitative thematic analysis using an iterative, inductive process.

**Results:**

Participants primarily expressed shared experiences, despite having unique caregiving situations. We identified themes among a) caregiver experiences: Trying to Do It All, Balancing Complex Emotions, Managing Expectations, and Adjusting to Changes Over Time and b) caregiver needs: Longing for Breaks and Self-Care; Lacking Help, Support and Resources; and Desiring Understanding and Recognition.

**Conclusions:**

These findings emphasize that many elements of the caregiving experience transcend care recipient age, condition, and relationship and are applicable to clinicians, researchers, and policy makers. The evidence of shared caregiver experiences can guide efficiencies in policy and practice (e.g., pooling of existing resources, expansion of interventions) to meet the needs of a broader population of caregivers.

## Introduction

Family caregivers are an integral part of the family system and care delivery team (National Alliance for Caregiving and American Association of Retired Persons, 2020). It is estimated that there are more than 250 million carers worldwide, including approximately 53 million family caregivers in the United States (US), providing care for adults or children with health care needs or a functional disability (Bose et al., 2021; International Alliance of Carer Organizations, 2021; National Alliance for Caregiving and American Association of Retired Persons, 2020). These individuals, also known as “family carers,” “informal caregivers,” and “close caregivers” (International Alliance of Carer Organizations, 2021), provide supportive care for family, friends, or neighbours with chronic health conditions and/or disabilities and do so out of relational obligation, rather than for professional purposes (Family Caregiver Alliance, 2021a; International Alliance of Carer Organizations, 2021). Family caregivers provide comprehensive and complex care in the community for individuals across the lifespan, making significant contributions to the health system both financially and instrumentally (Committee on Family Caregiving for Older Adults, 2016; National Alliance for Caregiving and American Association of Retired Persons, 2020; Rosalynn Carter Institute for Caregivers, 2020). At the population level, family caregivers in the US provided approximately $600 billion dollars of unpaid contributions in 2021 (Reinhard et al., 2023) and an estimated £162 billion pounds per year in the United Kingdom (Petrillo & Bennett, 2023).

The COVID-19 pandemic has both unveiled and amplified the challenges for family caregivers (Muldrew et al., 2022; National Rehabilitation Research and Training Center on Family Support, 2020; Reinhard et al., 2023) and underscored the valuable contributions informal caregivers make to society. The important role of family caregivers is gaining recognition in both clinical and policy environments, evidenced by initiatives and reports worldwide including the American Family Physicians (Swartz & Collins, 2019) and National Academy of Medicine (Schulz, 2016) in the US, the Department of Health in United Kingdom (Department of Health, 2008), and the Eurocarers’s “Enabling Caregivers to Care” initiative to empower caregivers throughout the European Union (Eurocarers, 2018). Multiple reports provide a population-based view of family caregiving, such as the NAC report on Caregiving in the U.S., the CDC report on caregiving, and the IACO’s Global State of Caring report (International Alliance of Carer Organizations, 2021; National Alliance for Caregiving and American Association of Retired Persons, 2020; National Association of Chronic Disease Directors, 2018). While these reports are valuable in recognizing quantifiable patterns of experience for family caregivers, they have not examined the lived experiences from the perspectives of family caregivers in the context of diverse health conditions, ages, and relationships.

Historically, family caregiver research has focused primarily on caregivers of older adults or has been siloed by the relationship between caregiver and care recipient (i.e., spouse/partner, parent/child) and/or by the health condition or disease process of the care recipient (Committee on Family Caregiving for Older Adults, 2016; Utz & Warner, 2022). Gaps remain in understanding the day-to-day challenges and concerns of caregivers’ experiences, which can be best understood through the stories and voices of caregivers (Agius, 2013). Additional caregiving work that moves beyond the siloes and specifically queries caregivers’ lived experiences is needed to provide a comprehensive and nuanced understanding of caregivers’ similarities and unique challenges across relationships, ages, and conditions (Fenton et al., 2022; Utz & Warner, 2022). Therefore, the purpose of the current study was to gain a deeper understanding of the similarities and differences of diverse family caregivers’ lived experiences across ages, caregiving relationships, and health conditions. Greater understanding of similarities in diverse family caregiver experiences can be used to guide centralized caregiver information (Stanford Distinguished Careers Institute, 2021), interventions, comprehensive community and national resources, and future health policy related to the needs of family caregivers.

## Materials and methods

The current qualitative study explored the perspectives of caregivers living in one US state caring for individuals of all ages and conditions. We chose a focus group approach for the study because we aimed to look across multiple caregiver experiences, and to elicit discussion among caregivers about similarities and differences in their experiences. The university Institutional Review Board approved the study procedures. All participants provided consent prior to participating and received $75 compensation for their participation. The research team contracted an experienced team from the University’s National Institutes of Health funded Clinical and Translational Science Awards (CTSA) Program, the Community Collaboration and Engagement Team (CCET) to manage recruitment and data collection for this project. The CCET maintains strong partnerships with community, university, and health department organizations in the area (University of Utah, n.d.).

### Participants

The research team and CCET collaborated to recruit participants using flyers distributed to local non-profit organizations that support caregivers (e.g., the Alzheimer’s Association local chapter, Utah Caregiver Coalition) as well as organizations that serve diverse communities across the state (e.g., Community Faces of Utah, Utah Health Department). Recruitment materials were designed to recruit a diverse group of caregivers, representing different caregiving relationships and disabilities/conditions. Inclusion criteria required that participants be adults (≥18 years of age) identifying as a primary informal or unpaid caregiver/care-partner (e.g., spouse/partner, parent, child, sibling) of an individual with a long-term disability and/or health condition requiring caregiving. There were no exclusion criteria.

#### Participant description

Our sample included 26 caregivers who had a variety of caregiving roles across relationships and conditions/diseases. Caregivers identified as caring for a parent, child, partner/spouse, sibling, or individual of other relation (e.g., niece, nephew, grandparent, in-laws) across a broad range of conditions including dementia, Parkinson’s disease, traumatic brain injury (TBI), multiple sclerosis, stroke, spinal cord injury (SCI), mental health conditions, Type 2 diabetes, and intellectual or developmental disability (IDD). The caregiving tenure spanned 5 months to 22 years. Caregivers estimated spending between 12–148 hours per week with the care recipient. All the caregivers reported assisting with medical management including attending appointments, taking medication, or following provider instructions, and 27% were providing care for more than one person with a long-term illness, injury, disability, and/or other condition. Table I describes the demographic characteristics of the participating caregivers: the majority identified as female (73%, *n* = 19), the mean age was 49.7 years (*SD* = 12.3), and just over half identified as non-Hispanic White (53.8%, *n* = 14). Most had some college education or higher (92%, *n* = 24).

**Table I.** Participant description.

Characteristic	N (%)
Age in years *Mean (SD)*	49.7 (12.3)
Gender	
Female	19 (73.1%)
Male	7 (26.9%)
Race and Ethnicity	
African American or Black	4 (15.4%)
Asian	1 (3.8%)
Caucasian or White	14 (53.8%)
Hispanic/Latino	3 (11.5%)
Pacific Islander	4 (15.4%)
Other (fill in: Creole Indian)	1 (3.8%)
Annual Household Income	
≥$50,000	15 (57.7%)
<$50,000	10 (38.5%)
Missing	1 (3.8%)
Highest Education Level	
High school	2 (7.7%)
Some college (including Associate degree)	10 (38.5%)
Bachelor’s degree	8 (30.8%)
Graduate degree	6 (23.1%)
Caregiver Relationship to Care Recipient^a^	
Caring for parent	8 (30.8%)
Caring for child	8 (26.9%)
Caring for spouse or partner	5 (19.2%)
Caring for sibling	3 (11.5%)
Other (fill in: grandparent, in-laws, niece, and nephew)	3 (11.5%)

^*a*^One caregiver was caring for recipients in more than one category (i.e., spouse and children), therefore, the total exceeds 100%.

### Data collection

Prior to the focus groups, all participants completed an online survey through the encrypted, HIPAA-compliant Research Electronic Data Capture (REDCap (Harris et al., 2009); web application including demographic information and details of their caregiving role (e.g., whether the caregiver lived with the care recipient, hours per week assisting care recipient, types of tasks). Using the demographic information, individuals were selected for invitation aiming for diversity of caregiver experiences and backgrounds within each group. All participants were also given an opportunity in the online survey to elect to be contacted again in the future for caregiver-related research and policy initiatives.

An experienced facilitator trained in focus group interviews conducted the data collection in the fall of 2020. The focus groups were conducted in a virtual setting (using Zoom) to adhere to COVID-19 precautions, as well as to maximize participation from caregivers who lacked transportation, lived in remote areas, or were unable to leave the care recipient. An additional benefit of using Zoom was that participants were given the opportunity to send the facilitator a private message with their responses if they felt uncomfortable saying them out loud. The focus groups followed a structured interview guide (Appendix A), ranged in size from 7–9 participants, and lasted approximately 2 hours. Additionally, two individual interviews using the same guide were conducted for participants whose care recipient had recently passed away.^1^

After each focus group and interview, participants were sent a brief survey to provide feedback on their experience with participating. In addition, the CCET sent each participant a document summarizing the discussion they participated in, and an opportunity to clarify or add anything they felt was missing. A team member (EW) transcribed the audio recordings of the focus groups and interviews verbatim. Participants were anonymized by assigning a letter based on the focus group they attended (i.e., A, B, C) and a randomly assigned number (i.e., 1–10).

### Data analysis

Four interdisciplinary team members (SN, MH, EW, AK) conducted a reflexive thematic analysis of the focus group transcripts using an iterative, inductive process (Braun & Clarke, 2006, 2021, 2022; Clarke & Braun, 2018). We centred the experiences and meaning participants ascribed to their experiences (e.g., essentialist or realist framework (Braun & Clarke, 2006), building our analysis on the basis that participants are experts on their own experiences. We followed Braun and Clarke’s (2022) six phases for reflective thematic analysis. Prior to initiating the coding process, we became familiar with the data through multiple readings of the transcripts and discussions in weekly team meetings (phase one). Each coder independently read and coded the data between meetings (phase two); initial team meetings involved discussing inductive codes. Team members then progressed to discussing initial themes and collated the coded data accordingly between meetings (phase three). In line with Braun and Clarke’s (2022) approach, developing, refining, and reviewing themes was an active and iterative process engaged in by the coding team (phases four and five). We used Dedoose (Version 8.3.45) analytic software to facilitate team coding and analysis (Dedoose, 2021); each coding team member reviewed each coded focus group to identify areas of uncertainty and further aid in refinement (phase five). The analysis team documented all coding decisions and processes of thematic generation. Any disagreements with coding and thematic analysis were resolved by consensus through iterative discussions throughout the process. The remaining authors reviewed the documentation three times during the course of analysis as an internal audit to enhance dependability of the process (Lincoln & Guba, 1985). To further enhance trustworthiness, we provide data extracts in the write-up (phase 6) to represent the findings (Braun & Clarke, 2021; Creswell, 2013).

## Results

**Figure 1.**
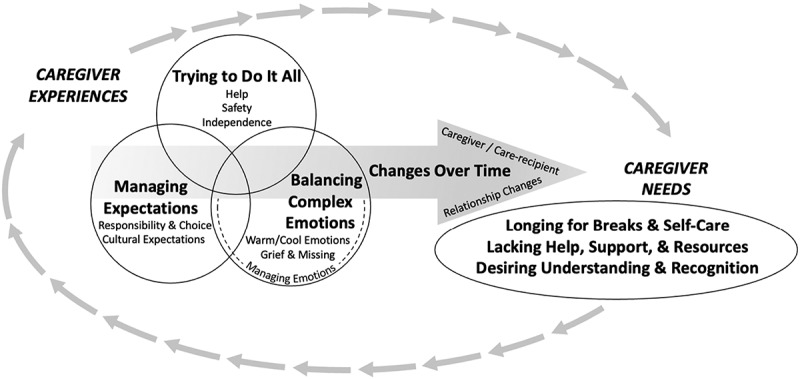
Caregiver experiences and needs across conditions, relationships, and the lifespan. Caregiver experiences include *Trying to Do It All*, *Managing Expectations*, and *Balancing Complex Emotions*—which all overlap and interact with one another, as displayed in a Venn diagram. These experiences are also depicted with an arrow indicating *Adjusting to Changes Over Time*—specifically, changes in the care-recipient, caregiver, and/or the caregiving relationship. All of these experiences inform and are informed by substantial caregiver needs including the need for: *Longing for Breaks and Self-Care*; *Lacking Help, Support, and Resources*; and *Desiring Understanding and Recognition* for the caregiver.

Analysis of the focus group data converged into two result domains: caregiver experiences and caregiver needs. *Caregiver Experiences* included four themes: (1) Trying to Do It All, (2) Balancing Complex Emotions, (3) Managing Expectations, and (4) Adjusting to Changes Over Time. *Caregiver Needs* encompassed three themes of (1) Longing for Breaks and Self-Care, (2) Lacking Help, Support and Resources, and (3) Desiring Understanding and Recognition. We integrated the findings into a visual representation of the relationship between the themes across conditions, relationships, and the life span, which is presented in Figure 1. Representative quotes are highlighted in Table II.

**Table II.** Qualitative themes with exemplar quotes.

Caregiver Experiences	
**Trying to Do It All**	
Help	“I provide financial assistance, administer medication, bathing, preparing meals, toileting, outing activities, doctor appointments and a safe environment.” (A3, Female, Creole Indian race, Caring for child)
Independence	“I’ve had to like pick him up, dust him off, and get him back on his horse, and tell him you gotta do it again” (C3, Female, Pacific Islander race, Caring for child) “The most important thing for me is trying to find the balance to be able to help her but yet not help her too much” (B7, Male, White race, Caring for partner/spouse)
Safety	“We just have to make her food because sometimes she’ll forget that the oven or the stove is on” (A8, Female, Pacific Islander race, Caring for parent) “I even have to get up a few times at night to check and make sure everybody’s not just in bed but also breathing and she hasn’t had a seizure in her sleep” (A6, Male, White race, Caring for spouse and children)
**Balancing Complex Emotions**	
Cool Emotions	“To watch a person just die you know a minute at a time those are the tough things and to watch her lose track of our three kids, their names, barely recognizes ‘em and our seven grandkids, I mean, those relationships have just disappeared” (A2, Male, Caucasian, Caring for partner/spouse) “I feel defeated…it’s hard being a caregiver and we may look like we have it together on the outside but for me emotionally and mentally, I was breaking down everywhere and hiding in corners that I never thought I could ever find” (C3, Female, Pacific Islander, Caring for child)
Warm Emotions	“Showing her love and knowing that although she couldn’t always understand it, that it still was important to love her and to care about her, that was the most important part.” (D1, Male, African American, Caring for sibling) “I know she’s happy I can tell she’s glad to be around family and that makes me happy, and I know I’m doing what I’m supposed to be doing” (A5, Female, Caucasian, Caring for sibling)
Grief	“I miss the young man he’d grown into being and what he, who he is now. You know I love him; he’s my son, but it’s very different” (C1, Female, Caucasian, Caring for child) “We were high school sweethearts, and you know we had all these plans for our lives…I thought at this point in our lives when we were younger, we’d be looking forward to retirement, going on vacations, our kids would be graduating from high school, I had no idea I’d be taking care of my family like this” (A6, Male, White race, Caring for spouse and children)
Managing Emotions	“I’m trying to make that as impersonal as I can because it’s just too painful, and I’ve also learned to try, and you know not let this thing take me down. […] I’m trying to survive myself. I want to have a life and a new sunrise if you will, beyond this thing, and those are some of the few things, probably the only thing that really gets me through day to day is knowing that maybe at the end of this thing I’m going to have a life and do some of the things that we no longer could, or you know punch my ticket on the bucket list that you know got set aside a long time ago” (A2, Male, Caucasian, Caring for partner/spouse) “I have a bigger sense of humour about this because if you don’t have a sense of humour about what’s going on, it’ll just tear you apart and it’ll all be stress.” (B9, Male, Caucasian, Caring for parent)
**Managing Expectations**	
Cultural Expectations	“I’ve always looked at it as a culture type of thing where we are expected to take care of our parents, it’s not wait till something happens to them. I’m the only girl in my family and so that’s, I’ve always been raised to this is what your role is, … it’s always it’s always been something that I always knew that I was going to do.” (A4, Pacific Islander, Female, Caring for parent)
Responsibility and Choice	“anyone’s who’s just doing this because out of duty and love and no one’s telling you to do it, you’re not getting help, that’s the thing, so it would be nice to have some sort of place that you can go because you chose to do this, not because somebody told you to do this.” (A5, Caucasian, Female, Caring for sibling) “My family, like my immediate family, my parents you know, my wife and kids, always knew that I was gonna be there for them.” (D2, Caucasian, Male, Caring for parent)
**Adjusting to Changes Over Time**	
Care Recipient Changes	“I’ve been watching it get harder and harder and it’s not going to stop until I don’t know when, maybe until she passes away” (B7, Male, Caring for partner/spouse)
Caregiver Changes	“It’s taught me a lot about empathy and compassion and if I really thought I had it before, I did not, and I could admit that to all of you, you know it I would never wish this upon anybody, but it’s taught me an awful lot and it’s made me care for others more.” (C1, Female, Caucasian, Caring for child)
Relationship Changes	“Our relationship has totally changed I mean it’s still loving and he’s so sweet he’s really a good person and good to be around, but it’s not anything like our relationship was before, and I miss him” (B4, Female, Caucasian, Caring for partner/spouse)
**Caregiver Needs**	
Longing for Breaks and Self-Care	“I could use a lot of time by myself just to sit on a rock somewhere contemplate my navel or do whatever you do ‘cause I haven’t been alone forever.” (Group A, Participant 9, Male, Caucasian, Caring for partner/spouse) “Well, the most helpful to me is to know where there are opportunities for respite,” (Interview, Participant D1, Other Race/Ethnicity, Male, Caring for sibling)
Lacking Help, Support, and Resources	“I’ll have to go to [a] support group just to figure out how to deal with my anxiety and to help him because I’m the mom” (B1, Female, Hispanic, Caring for child) “I don’t feel like there’s anything for my son with regard to any sort of day facility, nothing; and it frustrates me because I know he’s not the only person out there that’s like he is. There are places for him, with people that are elderly with Alzheimer’s and I’ve tried to get him into a social situation, so as far as research I think that we as a society need to understand that there are other needs outside of the everyday normal families and that there are people that do need help and the respite comes in the fact that, but it’s not just for the caregiver, it’s actually for the person being cared for because they deserve something outside of the caregiver.” (Group C, Participant 1, Caucasian, Female, Caring for child)
Desiring Understanding and Recognition	“Well, I wanna tell you guys that I have a sign on my door, my front door, that says, “a caregiver lives here,” and that’s because, like I said before, that I think people need to recognize all the work that the caregivers do, and you know the money ‘cause they’re not being paid.” (Group C, Participant 5, Caucasian, Female, Caring for parent)

### Caregiver experiences

#### Trying to do it all

Participants shared their background of how they became caregivers, the context of their caregiving situation with their care recipient, and the day-to-day caregiving tasks they performed. This theme, *Doing it All*, encompassed the many activities and responsibilities associated with helping the care recipient with activities of daily living (ADLs) and instrumental activities of daily living (IADLs), ensuring the care recipient’s safety, supporting the care recipient’s independence, and providing emotional and behavioural support—as one caregiver expressed, “pretty much the whole ball of wax” (A2). One caregiver of a parent with multiple comorbidities said, “I am also the chauffer, the secretary, the banker, the shopper, everything else that we do” (A4).

While each caregiver’s responsibility was unique, participants shared a range and variation in care tasks including assisting with ADLs/IADLs (e.g., dressing, bathing, medication, transportation, meal prep, scheduling or coordination, and taking the care recipient to appointments). Ensuring care recipient’s safety included preventing falls, avoiding accidents (e.g., fires from cooking), and providing reminders such as to eat or dress appropriately for the weather. Supporting the care recipient’s independence was achieved by providing encouragement, allowing them to perform as much of a task on their own as possible, and supporting growth and learning. Many caregivers discussed their need to juggle these caregiving duties across multiple care recipients or with other responsibilities of their life. One mother of a child with a SCI expressed, “we as caregivers are also working moms, dads, and working spouses that are trying to balance out our norm with everyday chores” (C3). For many participants, the COVID-19 pandemic instigated an increase in caregiving responsibilities. In contrast, other caregivers were grateful for the change of pace and felt they could work from home and be more effective with caregiving. One daughter-in-law expressed, “I still work full time, I go to college full time, and I have them both [mother-in-law with dementia and father-in-law post-stroke] full time, so the pandemic actually has been kind of a blessing for us … that I’ve been able to work from home” (A10).

#### Balancing complex emotions

Caregivers used an array of emotions to describe their experiences. It is important to note that these expressions of emotions were not mutually exclusive; they were often expressed simultaneously by a single caregiver. Caregivers acknowledged they “want the best” for the care recipient and care for them out of love, while they also recognized the frustration, exhaustion, sadness, and grief that accompany caregiving.

##### Cool and warm emotions

Many emotions could be categorized as cool (i.e., more negative) or warm (i.e., more positive). Of the 23 participants who expressed emotions, nearly all expressed both cool emotions (*n* = 21, 91.3%) and warm emotions (*n* = 20,86.9%). Cool emotions included expressions of sadness, exhaustion, frustration, guilt, worry, and stress and were expressed in relation to care recipient decline, relationship changes, and needs for breaks and self-care. One caregiver expressed, “burnout set in a long time ago” (A6), while another expressed, “I have to carry that burden of feeling guilty that I’m not doing better with [my son]” (B3). A parent of a child with autism expressed, “It takes an indescribable toll on the carer emotionally, physically, financially and mentally” (A1). Cool and warm emotions often were expressed together. Even amidst the stresses, one male participant caring for his wife with acquired brain injury and children with mental health conditions and IDD expressed, “I wouldn’t give this up for anything” (A6). Caregivers of individuals with improving health or development tended to express more warm than cool emotions. Seeing the care recipient happy often contributed to emotions of joy for the caregiver. One parental caregiver stated, “to know that your loved ones are cared for with love and care makes it all worth it” (C3). Other participants discussed the rewards of caregiving, including joy, personal satisfaction in their abilities as a caregiver to meet the care recipient’s needs, the opportunity to spend more time together, and gratitude for their own health, which enabled them to serve as a caregiver.

##### Grief

While grief is traditionally used to describe the emotional reaction that follows the loss or death of someone, caregivers commonly talked about how they grieve or miss how things were before the care recipient got sick or before there was an accident. In reference to her son with acquired brain injury, one mother said, “I miss the young man he’d grown into being and what he, who he is now is, you know I love him he’s my son, but it’s very different” (C1). Participants caring for individuals who were older or with worsening conditions were more likely to use language expressing grief. One spousal caregiver expressed, “grief is gone on for so long and some days you think you should be over it, by that, I should be over by now and it just doesn’t. I grieve daily for him” (B4). Another said, “we grieve what we don’t have any more, but you know we’re thankful for what we still have” (B2).

##### Managing emotions

Caregivers commonly spoke about the need to cope with or manage the complex emotions generated through the caregiving experience, and some offered specific strategies they had adopted to address the array of emotions. Several participants spoke of the challenges of knowing that things will get worse, while needing to stay positive on a day-to-day basis. One mother of a child with an IDD said, “I try to remind myself to celebrate everybody’s achievements and to compare him just to himself as opposed to other people” (A1). Others talked about how difficult it was to calm their frustrations towards the care recipient because, in many cases, the care recipient was not aware or did not understand that they were causing such stress. In contrast, a few caregivers expressed that humour was how they cope, as making light of the situation eased the pain or helped them maintain a positive attitude. Additionally, several caregivers attend caregiver support groups to help them manage their emotions.

#### Managing expectations

Caregivers described personal, cultural, and societal expectations for their caregiver role. Reflecting on whether caregiving was a responsibility or a choice, participants shared feelings of both internal and external pressures to be the caregiver. Words and phrases ranging from “duty,” “love,” “responsibility,” “I was the only one,” “I am her anchor,” “I do it willingly,” “I always knew I would do it,” to “I was expected to do it” describe the multifactorial set of influences that define the expectation to be a family caregiver. One male caregiver stated, “I don’t know if it’s duty or love but everybody in this group is doing what they have to do to get the job done” (A9). Another male caregiver said, “there’s a lot I have to deal with, but I do it willingly” (A6). Many described not wanting the care recipient to be in a care community, leading them to take on the caregiving role. One daughter described the love she had for her mother, which lead her to advocate to take her mother home to care for her.

Ethnicity, neighbourhood, nationality, and family cultures further defined the caregiving role and contributed to the caregiving experience. For example, one Hispanic participant described not feeling supported by her mother and sisters due to their differing cultural beliefs of the cause of her children’s mental illness and intellectual disability. One Pacific Islander participant described her caregiving role as “something I’ve always been expected to do” (A4). Additionally, a white male caregiver described his culture and upbringing “in a neighbourhood where we all took care of each other” (D2).

#### Adjusting to changes over time

When describing the caregiving role, caregivers reflected on the ways they had experienced changes over time. These included changes in the care recipient’s health status (e.g., decline or progress), personal changes (e.g., growth, learning), and changes in relationships (e.g., caregiver-care recipient relationship, outside relationships).

##### Care recipient changes.

Many caregivers used language and imagery describing the care recipient’s decline, which took an emotional toll on them and other family members. For some, the change in the care recipient’s health status was sudden, leading to the acute need for additional support, whereas others described a slowly evolving change in care recipient health. One spousal caregiver whose wife has Alzheimer’s stated, “this is a really crappy story and it’s not going to end good…I dread the next little while because of that” (A9). Another spousal caregiver expressed, “all of a sudden one day you wake up and you’ve got all these grey hairs on your face, and you know you don’t notice each one changing, and it’s exactly what it’s like taking care of a loved one with the with Alzheimer’s” (A2).

While most caregivers reflected on decline, one notable difference for a few caregivers was watching the care recipient make positive changes or improvements over time. One mother who was providing care to a child with a SCI said, “the most rewarding part of being a caregiver is watching him grow, watching him succeed and be able to accomplish goals that he’s set for himself and that have been set for him” (C3). Similar sentiments were expressed when care recipients were doing much better than anticipated, “defying the odds” (A1), or when they were able to set and achieve their own goals. In the event of care recipient progress, caregivers were able to move from a very hands-on care-giver role to more of a supportive role. In describing this transition, one caregiver said, “I’m not part of the decline, I’m part of growth as of now” (A1).

##### Caregiver changes

Several participants described how they personally changed during the caregiving journey. They used language such as learning, “figuring out how to make it work”, and “embracing the unexpected”. Examples of learning included a greater capacity for self-evaluation, identifying new coping mechanisms, broadening their perspectives, letting go of anger, transforming resentment to love, recognizing areas for growth, and empowering themselves by gathering resources and knowledge about the care recipient’s condition. One caregiver stated, “I’ve learned a lot more about my own areas for growth” (B3). Another caregiver said she’s benefitted by “learning to hold [her] temper and be patient” (B8).

##### Relationship changes

Caregivers also identified the way that their internal and external relationships changed because of caregiving. Internal relationships were defined as between the caregiver and care recipient. Many caregivers described this relationship as improving over time by growing closer, stronger, or more open. In contrast, others said they experienced greater conflict and their relationship had become more challenging over time, especially for those where the care recipient’s condition or prognosis declined or because the care recipient no longer recognized the caregiver due to cognitive decline. A husband caring for his wife with dementia said, the relationship “has gone from, you know, really husband and wife, best friend forever to just almost an empty shell that needs maintenance” (A2). Further, several caregivers mentioned the challenge of a perceived or real role reversal, in which the caregiver was required to perform activities or roles once fulfilled by the care recipient. One caregiver stated, “I feel like I’m the mom now. I’m not the daughter. I mean I’m still her daughter, but I feel like I’m the mom” (B2).

External relationships, defined as those outside of the caregiver and care recipient, commonly diminished over time. One caregiver said that caregiving was a “life changing occupation and interferes with friendships” (C1). Another caregiver discussed the strain caring for his mother had placed on his marriage leading to arguing and disconnectedness with his spouse; he stated, “there’s a lot of reward out of doing this but there’s a lot of strain on my shoulders and on my life and on my marriage and even with my children” (B8). Caregivers also described grief incurred through changing family dynamics when the care recipient was no longer able to perform the same role within the family or the caregiving role impacted other family members. One caregiver said, “what we did not fully understand is the impact on our own personal lives and the relationships with our children, grandchildren, and friends” (A10).

### Caregiver needs

The second area identified similar *Caregiver Needs* across care recipient age and condition. Caregivers expressed their struggle to balance the needs of the care recipient with their own needs. Caregivers described these needs in terms of three themes: 1) needing to care for themselves and take breaks, 2) having help, support, and resources for caregiving, and 3) receiving understanding—whether from family, the care recipient, friends, health care team members, or society at large—for the role they take on as caregivers.

#### Longing for breaks and self-care

It was common for caregivers to identify feeling burned-out as a caregiver, while still needing to find strength to keep going. Participants recognized the importance of self-care while acknowledging it was hard to find time for it. One parental caregiver said, “we need to try to find a way of recharging regardless of what we do or who we care for, we need to recharge because if we don’t recharge, the next day is going to be more and more and more” (C7). Another parental caregiver said she frequently neglects self-care but sees “a difference when I do take care of myself how well I take care of others as opposed to when I neglect myself. I do see a decline in you know in everything really” (A1). One caregiver of a parent with multiple conditions said caregiving “can be overwhelming, but you have to find a balance in taking care of yourself first so you can continue helping others” (A4).

Participants also recognized the need for and value in respite, or breaks. One caregiver reflected, “we all just need a break sometimes” (C6). While respite is frequently desired, caregivers described significant challenges of getting or taking respite—these were related to ineligibility for services (due to age or condition), the shutdown of some formal respite services due to the COVID-19 pandemic, a lack of awareness of the available respite resources or services in their community, or a lack of family/friend support that would allow them to get a break. One caregiver of a parent with Parkinson’s disease expressed, “COVID stay-at-home/work-from-home has dramatically changed and increased caregiving responsibilities as respite opportunities through adult day services have vanished” (B10). Caregivers also described not trusting others to effectively care for the care recipient, which was a barrier to them taking a desired and needed break. One participant caring for his mother with dementia said that he worries about her when he’s not there and asked, “is anybody else competent enough to care for [her] the way I care for [her]?” (B9).

##### Lacking help, support, and resources

Caregivers also described needing help in their caregiver role. One male participant said, “I’m personally thinking I’m the best caregiver [my wife] should ever have. I don’t know if that’s true, but if someone could help me with that it’d make everybody’s life better” (A9). Having to ask family to provide help was a challenge for many caregivers, although some described sharing caregiving roles with family members. Participants also described difficulties accessing available resources at the family, community, state, and national level. One participant caring for her mother with dementia expressed, “the health and well-being of the caretakers is very important ’cause we need to be healthy to take care of our person who’s disabled or ill and we should also be able to receive help from the government” (A8). Caregivers who had moved or relocated the care recipient discussed frustration in inconsistent state policies to provide caregivers with support financially or in navigating available resources. Several caregivers acknowledged the availability of resources for older adults but expressed frustration at the lack of resources for their care recipient. One spousal caregiver said, “What resources are out there? ‘Cause, we’re aware of a number of things; we’ve participated in a few things dealing with [my wife’s condition] … some of it’s applicable, a lot of it’s not at least for us in our circumstances” (B7). Finally, participants discussed having to leave the workplace, adapt hours due to caregiving responsibilities, or rely on the consistency of external caregivers to maintain employment, and how this was particularly challenging with changes in resources during the pandemic.

#### Desiring understanding and recognition

Caregivers desired for their role as caregivers to be understood and for their caregiving situations to be recognized for the work required on a day-to-day basis. One female caregiver described frequently being asked how the care recipient was but said others “really don’t ask me how I’m doing” (C5). A male caregiver expressed similar experiences and said, “there’s a tendency to not really give a lot of credit, support, or credibility, for that matter, to the caregivers” (A2). Caregivers also described judgements from others about the care recipient being “perfectly fine.” One mother of a son with anoxic brain injury said, “I’ve had people say to me, ‘oh he’s quite high functioning,’ and they’ve been with him for all of ten minutes and so they really do not get it whatsoever” (C1). A few caregivers attributed experiencing external relationship changes with their spouse, adult children, or friends to a lack of understanding of the caregiving role. One participant said his caregiving duties were “causing [my spouse and me] to argue a lot, and become very disconnected from each other, which is not what I need right now. I need her support, but at the same time I see where she she’s having the feelings of neglect” (B8). Other caregivers described feeling judged for their physical appearance, because so much work goes into caregiving. One caregiver said, “they assume that if we don’t have time to brush our hair it’s because we didn’t make time” (C3). Another caregiver said she has found “quite a disconnect between the medical world and the caregiver world” (C5) in which providers are unable to understand her reality and have a discussion in lay terms.

## Discussion

Caregivers play a critical role in the healthcare system, providing necessary—and often unrecognized—care for individuals with disabilities and illnesses throughout the lifespan. This study examined the shared experiences of diverse caregivers in the US across the spectrum of care recipient ages, health conditions, disabilities, and relationships. While existing caregiving literature has often centred on the unique experiences of caregivers of people with particular conditions or caregiving relationships, there is a need to examine the experiences of diverse caregivers (Bose et al., 2021). Despite having unique caregiving situations, participants expressed many similarities in experiences, such as performing many different types of tasks, managing the complexity of emotions associated with the caregiving role, and navigating the inevitable changes in relationships over time. They also identified a similar set of needs, including self-care and respite, help identifying support and resources in the community, and greater public awareness, understanding, and recognition of the caregiver role. These results emphasize that many elements of the caregiving experience transcend care recipient age, health condition, and relationship. These important findings can help guide healthcare professionals and researchers to develop appropriate interventions, education, research, and policy initiatives that address these similar needs across caregivers.

Nearly all participants expressed the need for help with identifying available caregiver support resources. This is consistent with global research showing many informal caregivers have unmet supportive care needs and face challenges in obtaining access to community services, respite care, support groups or individual counselling (Cahill et al., 2023; Mollica et al., 2020; Muldrew et al., 2022). Consistent with prior research (Law et al., 2021), many participants in the study were unaware of the resources that are available to meet their needs, and several felt that the support services were geared only towards ageing-related services, which were inapplicable to many of their care recipients. Caregivers in this study also experienced employment stress when trying to do it all, which is similar to other findings in the literature demonstrating caregivers of all ages experience employment stress (Koumoutzis et al., 2021). These findings suggest caregivers may benefit from workplace programmes and policies designed to support informal caregivers (Koumoutzis et al., 2021). Furthermore, healthcare professionals can play a key role in both connecting family caregivers to resources within their communities, while also advocating for policies that support and protect the needs of family caregivers at a national level (Cahill et al., 2023). In the US, state policies for caregiver resources are varying and continually changing, and this inconsistency has led to uncoordinated efforts to support caregiver needs (Family Caregiver Alliance, 2021; Raj & Singer, 2021). However, the increasing importance of providing caregiver resources has recently received national attention in the US as evidenced by President Biden’s agenda to support informal caregivers through tax credits and expanding long-term care access, which has potential to increase awareness and availability of caregiver support services (Democratic National Committee, 2020). Globally, many countries including Sweden, Ireland, UK, and Canada, have national strategies to support and protect the health and well-being of informal caregivers (Cahill et al., 2023; Canadian Home Care Association, n.d.; Department of Health, 2008). Additionally, the French Association of Carers employs café-like environments to engage caregivers in an opportunity to share, learn, and exchange with other carers and professionals to gain respite, connection, social and emotional support (International Alliance of Carer Organizations, n.d.).

Participants in our study expressed juxtaposing warm and cool emotions and the complexity of managing those emotions. These findings across caregiver ages and roles are similar to reports indicating four in ten caregivers experience emotional strain (National Alliance for Caregiving and American Association of Retired Persons, 2020). Previous research demonstrates informal caregivers have multidimensional physical and mental burden, including worse mental health outcomes than non-caregivers (Hopps et al., 2017; Koumoutzis et al., 2021; Trivedi et al., 2014). Many informal caregivers face personal challenges such as financial impacts, worsening stress, changes in social and familial relationships, increased risk for chronic physical and mental health conditions, and greater mortality risk (Committee on Family Caregiving for Older Adults, 2016; DePasquale et al., 2017; National Alliance for Caregiving and American Association of Retired Persons, 2020; National Rehabilitation Research and Training Center on Family Support, 2020; Perkins et al., 2013; Reinhard et al., 2008). Despite these challenges, many caregivers also report positive experiences, such as gaining a sense of purpose or meaning, greater resilience, and personal growth as a result of caregiving (Harmell et al., 2011; Leipold et al., 2008; National Alliance for Caregiving and American Association of Retired Persons, 2020), which supports the need to increase national and international efforts to promote caregiver well-being. In the US, organizations like the National Alliance for Caregiving (2023) are leading efforts to address the population-level and individual mental health needs of caregivers by promoting mental health research, advocating for behavioural health equity, promoting healthcare provider readiness through training to address mental health needs, and supporting models to support the holistic needs of family caregivers. Nonprofit organizations like HelpGuide (2023) provide online access to mental health resources and address topics specifically for family caregivers. Internationally, advocating for the well-being and mental health of caregivers is a top priority for the International Alliance of Carer Organizations (2021).

Participants also described the need to be acknowledged and appreciated as caregivers, both socially by friends and family and also by members of the health care team, which is consistent with previous studies (Law et al., 2021). Previous research has demonstrated that social support improves psychological distress for carers, indicating increasing awareness of the caregiving role and interventions to foster perceived social support can be instrumental in reducing carer distress (George et al., 2020). Within the US, a few public awareness campaigns have been launched regionally and nationally to help caregivers self-identify in their role and illuminate the responsibilities of family caregivers (AARP, 2013; Cordano et al., 2015). In the US, the provisions in the CARE Act to identify a primary caregiver in a patient’s medical record and to include the family caregiver in hospital discharge planning is an important formal recognition of the role that family members play in the delivery of care that occurs in home and community-based settings (AARP, 2021). Global initiatives to improve the well-being of informal caregivers also include recommendations to raise awareness of the importance informal caregivers for society (International Alliance of Carer Organizations, 2021; Petrillo & Bennett, 2023; United Nations Economic Commission for Europe Working Group on Aging, 2019). Healthcare professionals should be aware of these policies to promote integrated health for family caregivers and ensure carers are included in as a vital part of the healthcare team for the care recipient (Embracing Carers, 2021; International Alliance of Carer Organizations, 2021; Law et al., 2021; Reinhard & Young, 2016). While these campaigns are a crucial first step to elevate the conversation about caregiving and increase recognition of family caregivers in the health care setting, additional emphasis should be placed on identifying and addressing the hidden needs of family caregivers as caregivers often neglect their personal well-being to focus on the needs of care recipients (Physician Data Query Supportive and Palliative Care Editorial Board, 2019). A formal caregiver assessment could gather information about the caregiver’s health and well-being and their educational and supportive care needs within their caregiving role. Identifying these needs provides opportunities for referrals for both caregivers and care recipients (Swartz & Collins, 2019) and recognizes the valuable role of the family caregiver (Reinhard & Young, 2016).

Though there were many similarities identified among the participants, there were some differences, which are related to the multidimensional aspects of caregiving. Differences were primarily related to caregiver or care recipient demographics, care recipient condition, uniqueness of the caregiving situation (i.e., caregivers’ tasks differed based on care recipient needs), and changes in the care recipient’s condition over time (e.g., slow vs. abrupt changes, decline vs. improvement). For most participants, especially those caring for someone with a progressive or terminal condition, improvement in the care recipient’s status and functioning was not an option and was not considered a goal or expectation, whereas others such as those caring for children with chronic health or disability, were guided more towards healing, rehabilitation, and/or development of independence as their primary goal. Awareness of these fundamental differences in caregivers’ experiences is important, as caring for a recipient with a progressive condition, assisting a care recipient with multiple ADL/IADL tasks, and/or having low perceived social support over time are all associated with increased caregiver burden (Lopez Hartmann et al., 2019; Riffin et al., 2019; Tramonti et al., 2019). These differences are a reminder that while caregivers do share many similarities in their experiences and needs, they also have unique challenges related to their specific circumstances and acknowledging these differences facilitates recognition of the caregiving role.

### Implications

The themes identifying caregiver needs can be used to inform actionable outcomes (Braun & Clarke, 2021). The findings point to several opportunities for healthcare professionals. One opportunity is to acknowledge caregivers as members of the healthcare team, recognizing the critical role that they play while also typically being unsupported (Law et al., 2021). Another is for professionals to provide support and condition-specific anticipatory information to both clients and caregivers to help effectively provide care for clients. Critically, professionals should also seek out opportunities to provide resources and support directly to the caregiver, such as support groups or other psychosocial resources. All these aspects of working with and supporting caregivers should be integrated into professional training programmes for healthcare providers.

Evidence of shared experiences and needs identified across conditions and relationships can guide professionals and researchers in the pooling of resources and direct expansion of interventions to meet a broader population of caregivers, rather than focusing on resources and support restricted to ageing services. For example, participants in this study would have welcomed an easily accessible directory providing comprehensive information about local and web-based caregiver resources that span care recipient age and health condition(s) such as the community resources provided by Caregivers Alberta (2023). Healthcare providers can recommend mobile resources such as the Jointly app created by CarersUK to help informal caregivers stay organized with care-related tasks and create a circle of care for the care recipient (Carers, 2023). Caregiver support groups can utilize findings from this study to focus on the similar needs of caregivers while also recognizing the individual’s unique circumstances and caregiving needs. As caregivers continue to report decline in their own health status (National Alliance for Caregiving and American Association of Retired Persons, 2020), interventions should address not only the care recipient’s needs but also the caregiver’s physical and mental health needs. Innovative tools and approaches are needed to screen, assess, and treat caregivers’ unique mental health needs (National Alliance for Caregiving, 2023). Healthcare professionals are well positioned to develop and provide holistic, caregiver-focused interventions which should include components of emotional and psychosocial support, a resource repository of physical and financial assistance programmes, medical guidance to facilitate condition-specific education and anticipatory guidance, and tools to successfully navigate the healthcare system (Reinhard & Young, 2016). Future interventions centred on the similarities of caregiving could focus less on performing the physical tasks related to a specific disease state (e.g., ADLs) and more on the findings of this study including managing the complexity of emotions that caregivers frequently encounter with the caregiving role, navigating potential relationship changes over time, and identifying support and resources in the community related to caregiving and respite.

### Limitations and strengths

The findings of this study should be viewed in light of the limitations. Because we collected data predominantly in group format, we may not have captured the depth of the caregivers’ experiences that we could have if we had conducted one-on-one interviews. Further, participants were potentially influenced by the group dynamics and may have censored or withheld comments that would have been stated in an individual interview (Carey, 2016). However, in a post-participation survey, participants expressed gratitude for the focus group, which helped them realize there were other caregivers in similar situations and that they were not alone. They also commented that the focus group facilitator orchestrated the group well and gave participants ample time to express their views and open up. Despite being given the opportunity to privately share their responses with the facilitator, no participants shared private responses or expressed feeling like they could not share their experiences within the group. Additionally, as caregivers are busy and frequently juggle multiple responsibilities, the video-conferencing focus groups were an ideal way to engage participants in a meaningful discussion with other caregivers, which highlighted their similarities. Using the online focus group format met participant needs by allowing them to stay at home without needing to find alternative care for the care recipient and addressing their concerns about the safety of meeting during the COVID-19 pandemic. Finally, while the participants were heterogeneous in terms of their race, ethnicity, socioeconomic status, and caregiving experiences, all participants were living in one state of the US, and 92% had some college education, which may limit relevance to other groups of diverse caregivers with differing levels of education and access to caregiving resources. However, this study recruited diverse caregivers with a broad range of experiences, which added to the breadth of the findings and literature regarding the commonalities of family caregivers across the lifespan regardless of care recipient health condition.

## Conclusion

The results of the shared experiences of caregivers in the US add to the growing body of international evidence that caregivers need more support, resources, and increased recognition. This research expands our understanding of informal caregivers’ similar experiences cutting across diverse caregiving situations with regard to disabilities, health conditions, age, and caregiving relationship. One participant summed up the caregiver similarities by stating, “I don’t really see a big difference. I mean everybody’s kind of got somebody different to take care of and everything is slightly different, but in my opinion, for the most part we all carry the same burden, we all carry the same worries, the same grief” (B8). While all caregivers are unique, understanding the many commonalities that exist across caregiver experiences can help healthcare professionals identify efficient ways to address the needs of family caregivers through future policy and research interventions.

## Data Availability

Raw data, including audio recordings and transcripts, are not available to other researchers for replication purposes as we did not obtain written informed consent from the participants to share their raw data. However, to promote transparency, we have included the interview guide and final codebook detailing the codes, categories, and example texts.

## Note

1. Two individuals with recently deceased care recipients were interested in participating. However, our team was hesitant to include grieving participants in the focus group environment with current caregivers. We used individual interviews with these participants to allow them to share their perspectives without altering the focus group experience for others.

## Appendix A

Semi-Structured Focus Group Guide

1. We will go around the group and ask each of you to introduce yourself and describe your role as a caregiver/care-partner for your family member. Please briefly explain the following:

a. What is the nature of your relationship (e.g., spouse, daughter, friend) and what kind of conditions (e.g., diabetes, dementia), and/or disabilities (e.g., vision impairment, immobility) does your family member have?

b. How long have you been in a caregiving/care-partner relationship and how has the nature of your relationship changed over time?

c. What kind of care do you provide to your family member?

2. In our experience, caregivers often say there are both challenges and rewards associated with caregiving. How would you describe your experience of being a caregiver/care-partner?

a. What are the most rewarding parts of being a caregiver? What are the most challenging aspects of being a caregiver?

3. What similarities and differences do you see between yourself/your experiences as a caregiver and experiences of other caregivers/care-partners? This could include others here today in this group or others outside of this group (e.g., friends, neighbours, other family members).

a. If needed, prompt: in healthcare and research, it is sometimes assumed that people identify most strongly as a caregiver/care-partner of someone with a certain type of condition/illness; for example, a dementia caregiver. However, people may also identify more with their caregiver/care-partner relationship, such as “parent,” “adult child,” “spouse.” Or they may identify with other aspects of their caregiving/care-partnering experience. How do you identify as a caregiver? What is the most important part(s) of your identity as a caregiver?

b. Follow-up prompt: Where do you find or get support for yourself as a caregiver? Give examples (as necessary) such as websites for specific foundations associated with illness or disability, friends, other families who are providing care.

4. Over the past several months, the COVID-19 pandemic has changed many things about daily life. How has the COVID-19 pandemic affected your caregiving experience?

5. So that our research captures your experiences and meets your needs, what can you tell us about what’s most important to you in terms of caregiving? What do you think research should focus on?
